# Supplementary material for: Last-mile delivery increases vaccine uptake in Sierra Leone
Source: Nature. 2024 Mar 13;627(8004):612–9. doi: 10.1038/s41586-024-07158-w (PMC10954551; doi:10.1038/s41586-024-07158-w)
Supplement: Supplementary file 1 — Supplementary Information [file 41586_2024_7158_MOESM1_ESM.pdf]

nature portfolio

https://doi.org/10.1038/s41586-024-07158-w

Supplementary information

Last-mile delivery increases vaccine uptake in Sierra Leone

In the format provided by the authors and unedited

Supplementary Information: Last-Mile Delivery Increases Vaccine Uptake in Sierra Leone

Niccolò F. Meriggi[1,2,3] Maarten Voors[2] Madison Levine[4]
Vasudha Ramakrishna[5] Desmond Maada Kangbai[6] Michael Rozelle[2]
Ella Tyler[2] Sellu Kallon[2,7] Junisa Nabieu[2] Sarah Cundy[8]
Ahmed Mushfiq Mobarak[9*]

[1]International Growth Centre, Freetown, Sierra Leone

[2]Wageningen University & Research, Wageningen, the Netherlands

[3]Centre for the Study of African Economies, University of Oxford, Oxford, United Kingdom

[4]University of Illinois, Urbana, Illinois, United States of America

[5]Boston University, Boston, Massachusetts, United States of America

[6]Ministry of Health & Sanitation, Freetown, Sierra Leone

[7]University of Sierra Leone, Freetown, Sierra Leone

[8]Concern Worldwide, Freetown, Sierra Leone

[9]Yale University and Y-RISE, New Haven, United States of America

[*]Corresponding author. E-mail: ahmed.mobarak@yale.edu.

December 22, 2023

Contents

1 Additional Figures **2**

1.1 Consort Diagram 2

1.2 Variation in Endline Vaccination Rate 3

1.3 Variation in Number of People Vaccinated in Each Community 4

1.4 Variation in Number of Vaccines Administered per Community by Vaccination Team 5

1.5 Effect Sizes in Other Vaccine Uptake RCTs 6

1.6 Interaction Effects with demographic variables 7

2 Literature Review **8**

1. Additional Figures

1.1 Consort Diagram

Figure A1: Consort Diagram

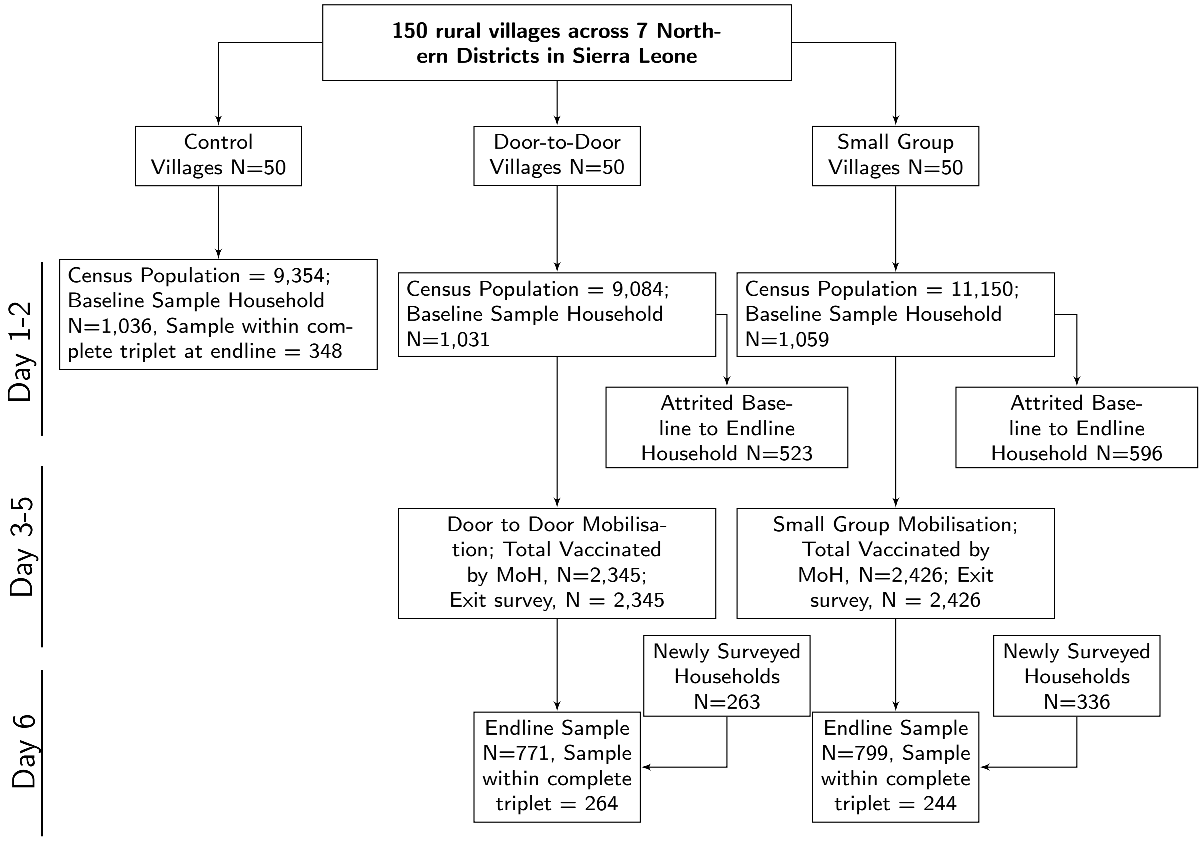

1.2 Variation in Endline Vaccination Rate

Figure A2: Variation in Endline Vaccination Rate

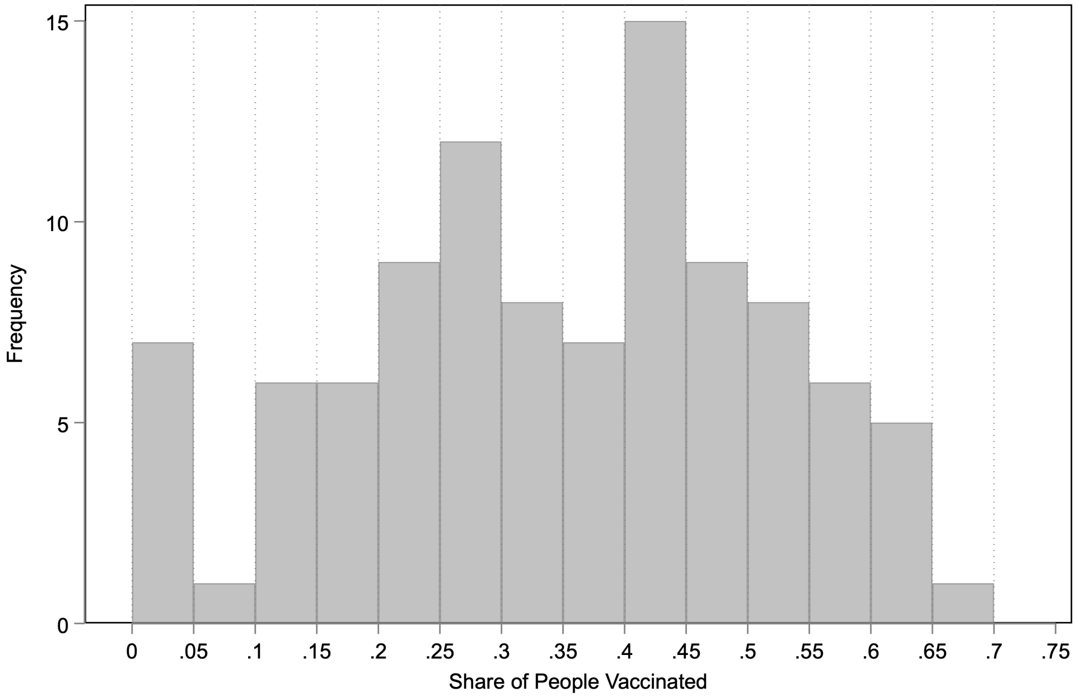

Notes: This figure provides a histogram of the vaccination rate (i.e share of adults that took the vaccine at the end of the study from those enumerated during the census) in the 100 treatment villages.

1.3 Variation in Number of People Vaccinated in Each Community

Figure A3: Variation in Number of People Vaccinated in Each Community

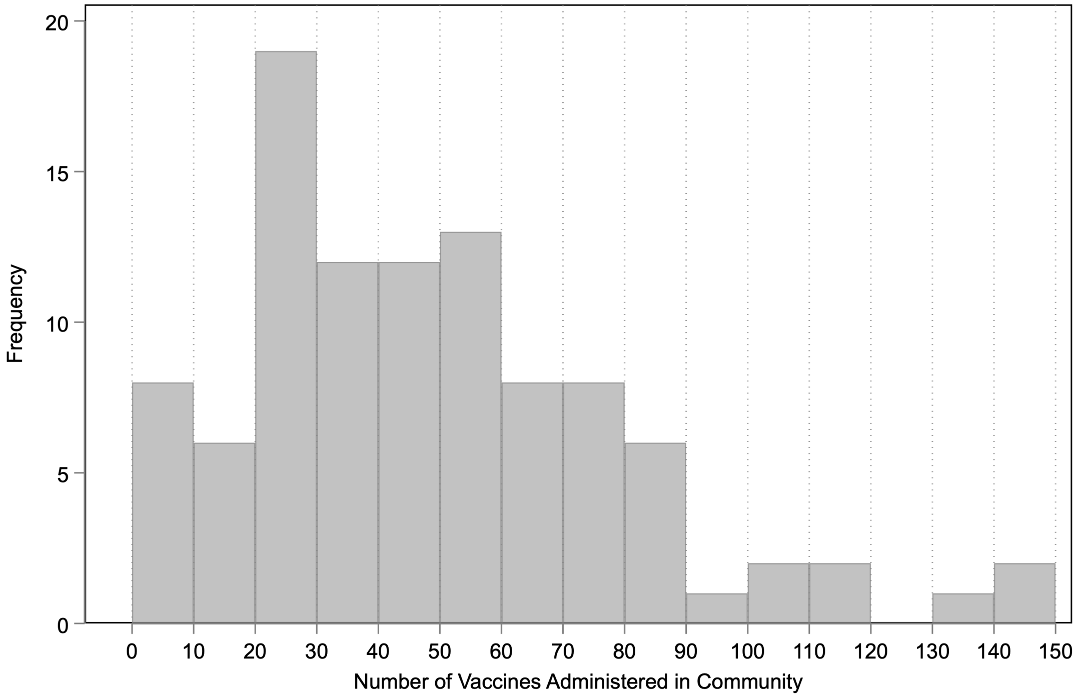

Notes: This figure provides a histogram of the number of vaccines administered in the 100 treatment villages.

1.4 Variation in Number of Vaccines Administered per Community by Vaccination Team

Figure A4: Variation in Number of Vaccines Administered per Community by Vaccination Team

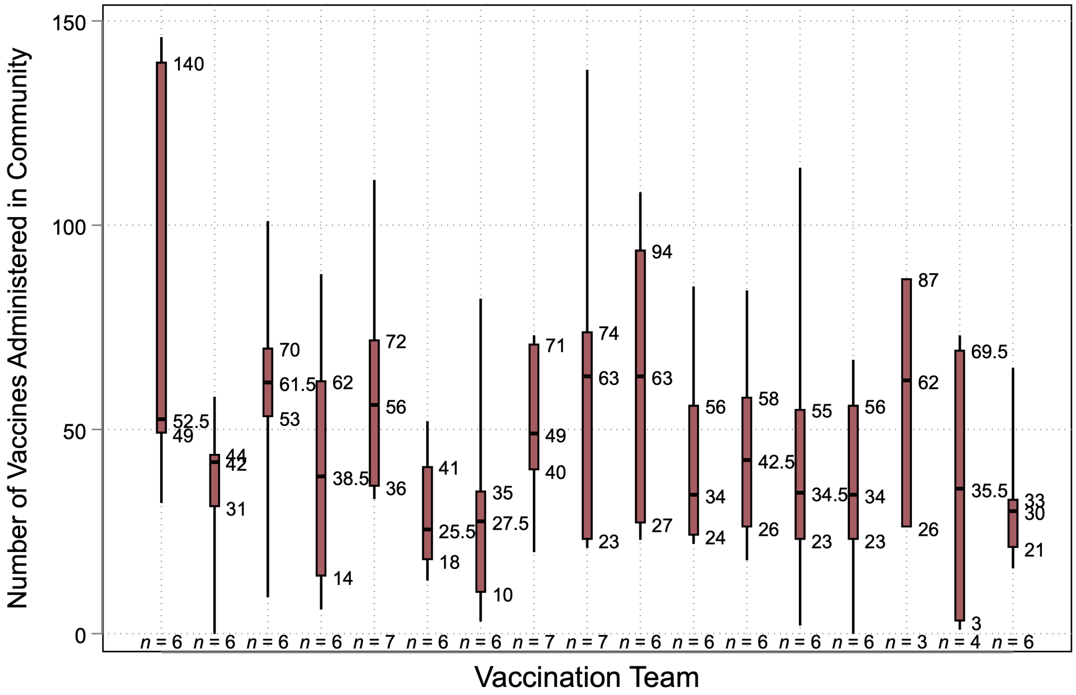

Notes: The box plots in this figure each represent the distribution of the number of vaccines administered in different communities by one COVID-19 vaccination team. The center line of each box plot represents the median number of vaccines that each team administered in a community. The top of each box represents the 75th percentile of the number of vaccines each team administered in a community, while the bottom of the box represents the 25th percentile of the number of vaccines each team administered in a community. The whiskers extend to the maximum and minimum values.

1.5 Effect Sizes in Other Vaccine Uptake RCTs

Figure A5: **Effect Sizes in Other Vaccine Uptake RCTs**

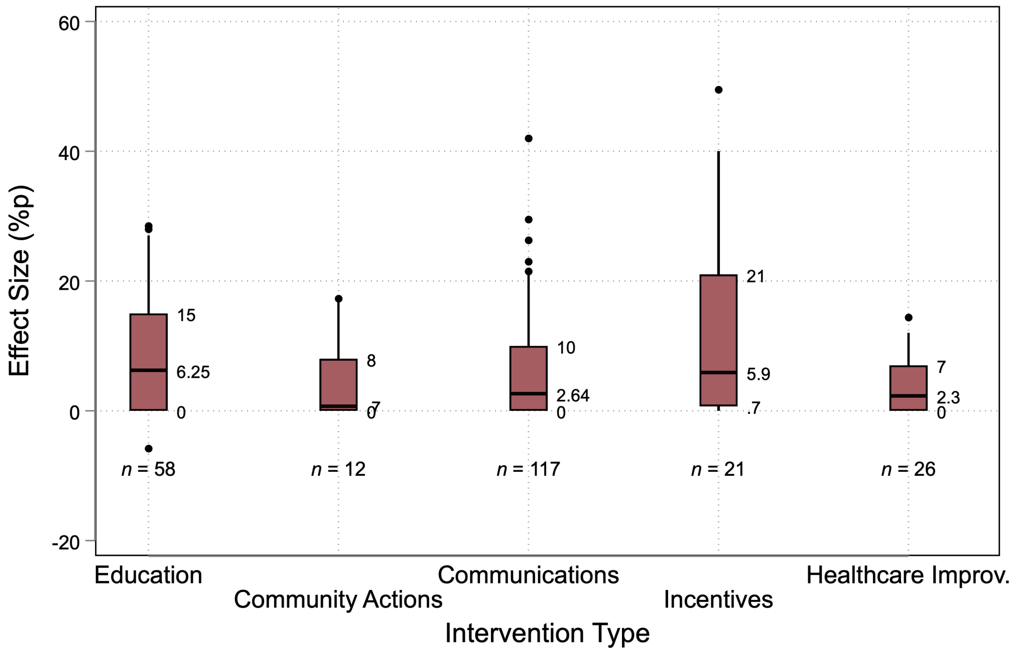

The figure shows boxplots with the percentage-point change in reported vaccine uptake relative to control group across the 234 treatments assessed as part of our literature review (see Table A1 in Supplementary Information Section 2). This includes a sizeable group of treatments for which there was no significant effect of the intervention (82 treatments, constituting 35% of all interventions reviewed). Effect sizes are summarized over five broad types of interventions. Each box represents the Interquartile Range (IQR), horizontal line is the median, whiskers indicate the 5th to 95th percentile, whilst outliers beyond these extremes are indicated with a marker.

1.6 Interaction Effects with demographic variables

Table A1: **Intent-To-Treat Effect of pooled treatment and interactions with demographic variables**

	(1)	(2)	(3)	(4)	(5)
Pooled Treatment	0.298***	0.311***	0.261***	0.362***	0.388***
	(0.020)	(0.027)	(0.019)	(0.031)	(0.028)
Female	-0.018**				
	(0.009)				
Female # Treatment	-0.067***				
	(0.016)				
Aged 18-24		-0.047**			
		(0.018)			
Aged 18-24 # Treatment		-0.122***			
		(0.028)			
Aged 25-54		-0.018			
		(0.013)			
Aged 25-54 # Treatment		-0.039*			
		(0.022)			
HH head has any schooling			0.007		
			(0.012)		
HH head educated # Treatment			-0.003		
			(0.018)		
HH owns land				0.038*	
				(0.022)	
HH owns land # Treatment				0.038	
				(0.035)	
HH ate reduced food portions					0.023
					(0.019)
Reduced food # Treatment					-0.006
					(0.032)
Mean in Control	0.062	0.062	0.062	0.062	0.062
No. of Observations	12096	12096	12096	2674	2674
No. of Villages	150	150	150	149	149
R^2	0.1411	0.1463	0.1341	0.2152	0.2115

The table presents Intent-To-Treat estimates of the pooled treatment and interactions with demographic variables. Dependent variable is the vaccination status at the end of the study of adults enumerated during the census. Regressions include randomization fixed effects (ie triplets), with heteroskedasticity-robust standard errors clustered at the village level. ***, **, and * indicate significance at the 1, 5, and 10 percent critical level.

2. Literature Review

Table A2: Effect Size of Previous Vaccine Uptake Studies

Intervention Type	Country of Study	Vaccine(s) Studied	Author	Publication Year	Effect Size (in %P)
Communications	USA	Immunization series completion by 24 months	Dini et al.	2000	8.3
Communications	USA	Influenza; Pneumococcal	Krieger et al.	2000	21.1
Communications	USA	Childhood immunizations	Vivier et al.	2000	12.5
Communications	USA	Childhood immunizations	Vivier et al.	2000	21.5
Communications	USA	Childhood immunizations	Vivier et al.	2000	14.8
Communications	UK	Influenza	Arthur et al.	2002	6.4
Communications	USA	Influenza	Ahmed et al.	2004	4
Communications	Spain	Tetanus-diphtheria	Ibáñez-Jiménez et al.	2007	18.3
Communications	USA	Pneumococcal (elderly)	Winston et al.	2007	9
Communications	USA	Pneumococcal (chronic)	Winston et al.	2007	10
Communications	USA	Influenza	Doratotaj et al.	2008	0
Communications	Israel	Influenza	Abramson et al.	2010	26.3
Communications	USA	Influenza	Chapman et al.	2010	12
Communications	USA	Influenza	Minor et al.	2010	13
Communications	USA	Influenza	Minor et al.	2010	23
Communications	USA	Influenza	Humiston et al.	2011	42
Communications	USA	Influenza	Milkman et al.	2011	4
Communications	USA	Meningococcus; Pertussis; TDAP	Szilagyi et al.	2011	14.3
Communications	USA	HPV initiation	Szilagyi et al.	2011	15.6
Communications	USA	HPV third dose	Szilagyi et al.	2011	12.4
Communications	USA	HPV second dose	Szilagyi et al.	2011	15.8

Intervention Type	Country of Study	Vaccine(s) Studied	Author	Publication Year	Effect Size
Communications	USA	TDAP	Szilagyi et al.	2011	12.1
Communications	USA	Influenza	Dombkowski et al.	2012	6.5
Communications	USA	At least one of: TDAP; HPV; Meningococcal conjugate	Suh et al.	2012	12.5
Communications	USA	All of: TDAP; HPV; Meningococcal conjugate	Suh et al.	2012	11
Communications	USA	Herpes zoster	Hess	2013	1.88
Communications	USA	Influenza	Moniz et al.	2013	0
Communications	USA	Meningococcus; Pertussis; HPV	Szilagyi et al.	2013	2
Communications	USA	Meningococcus; Pertussis; HPV	Szilagyi et al.	2013	5
Communications	USA	Childhood immunizations (7-month olds)	Dombkowski et al.	2014	0
Communications	USA	Childhood immunizations (12-month olds)	Dombkowski et al.	2014	0
Communications	USA	Childhood immunizations (19-month olds)	Dombkowski et al.	2014	7
Communications	USA	HPV completion	Patel et al.	2014	0
Communications	Zimbabwe	OPV1, Penta1 and PCV1	Bangure et al.	2015	20
Communications	USA	HPV completion	Chao et al.	2015	9.8
Communications	Beirut	Pneumococcal	Ghadieh et al.	2015	6.1
Communications	Beirut	Pneumococcal	Ghadieh et al.	2015	15.4
Communications	Beirut	Pneumococcal	Ghadieh et al.	2015	4.6
Communications	USA	Influenza	Hofstetter et al.	2015	3.7
Communications	USA	Influenza	Hofstetter et al.	2015	0

Intervention Type	Country of Study	Vaccine(s) Studied	Author	Publication Year	Effect Size
Communications	USA	All necessary vaccines	O'Leary et al.	2015	3.1
Communications	USA	Any vaccination	O'Leary et al.	2015	5.2
Communications	USA	Influenza	Stockwell et al.	2015	3.7
Communications	China	Full immunisation (5 vaccines)	Chen et al.	2016	0
Communications	UK	Influenza	Herrett et al.	2016	0
Communications	Netherlands	Influenza	Lehmann et al.	2016	0
Communications	Netherlands	Influenza	Lehmann et al.	2016	0
Communications	Bangladesh	BCG; Penta3; MR	Uddin et al.	2016	29.5
Communications	USA	Childhood immunizations	Glanz et al.	2017	0
Communications	USA	Childhood immunizations	Glanz et al.	2017	5.9
Communications	USA	Eligible child receiving any vaccine	Kempe et al.	2017	5
Communications	Australia	Influenza	Regan et al.	2017	3
Communications	USA	Influenza	Yudin et al.	2017	0
Communications	Australia	Influenza	Borg et al.	2018	1.5
Communications	USA	Influenza (high-risk adults)	Hurley et al.	2018	0
Communications	USA	Influenza (over-65s)	Hurley et al.	2018	3.4
Communications	USA	Influenza (no high risk adults)	Hurley et al.	2018	0
Communications	USA	Influenza	Yokum et al.	2018	.9
Communications	USA	HPV	Dempsey et al.	2019	0
Communications	USA	Influenza	Nehme et al.	2019	0
Communications	USA	Influenza	Nehme et al.	2019	2.5
Communications	USA	Influenza	O'Leary et al.	2019	19
Communications	USA	Influenza	O'Leary et al.	2019	21

Intervention Type	Country of Study	Vaccine(s) Studied	Author	Publication Year	Effect Size
Communications	USA	TDAP	O'Leary et al.	2019	0
Communications	USA	TDAP	O'Leary et al.	2019	0
Communications	USA	Pneumococcal vaccination; herpes zoster	Stolpe et al.	2019	0
Communications	Australia	HPV	Tull et al.	2019	2.64
Communications	Australia	HPV	Tull et al.	2019	3.29
Communications	USA	Influenza; Tetanus; Shingles; Pneumonia	Chen et al.	2020	.27
Communications	USA	Influenza; Tetanus; Shingles; Pneumonia	Chen et al.	2020	.06
Communications	USA	Influenza; Tetanus; Shingles; Pneumonia	Chen et al.	2020	.15
Communications	Nigeria	Childhood immunizations	Kawakatsu et al.	2020	5
Communications	USA	Influenza	Lee et al.	2020	1.2
Communications	Hong Kong	Influenza	Liao et al.	2020	0
Communications	USA	HPV initiation (New York)	Szilagyi et al.	2020	0
Communications	USA	HPV initiation (Colorado)	Szilagyi et al.	2020	2.3
Communications	USA	Influenza	Wijesundara et al.	2020	1.3
Communications	USA	Influenza	Wijesundara et al.	2020	0
Communications	France	Influenza	Barbaroux et al.	2021	0
Communications	Canada	HPV	Bernard-Genest et al.	2021	0
Communications	USA	COVID-19	Dai et al.	2021	3.57
Communications	USA	HPV series intitiation	Gerend et al.	2021	12.8
Communications	USA	HPV completion	Gurfinkel et al.	2021	0
Communications	USA	HPV initiation (New York)	Gurfinkel et al.	2021	0

Intervention Type	Country of Study	Vaccine(s) Studied	Author	Publication Year	Effect Size
Communications	USA	HPV initiation (Colorado)	Gurfinkel et al.	2021	0
Communications	USA	HPV completion	Gurfinkel et al.	2021	0
Communications	USA	HPV initiation (Colorado)	Gurfinkel et al.	2021	0
Communications	USA	HPV initiation (New York)	Gurfinkel et al.	2021	0
Communications	USA	HPV completion	Gurfinkel et al.	2021	0
Communications	USA	HPV completion	Gurfinkel et al.	2021	1.4
Communications	Kenya	Measles	Kagucia et al.	2021	10
Communications	USA	Influenza; Pneumococcal; TDAP	Kappes et al.	2021	0
Communications	USA	Influenza	Kappes et al.	2021	0
Communications	USA	Influenza	Kappes et al.	2021	0
Communications	USA	Numerous	Kappes et al.	2021	.3
Communications	USA	Influenza	Kappes et al.	2021	0
Communications	USA	Influenza	Kappes et al.	2021	.6
Communications	Ghana	Polio; BCG	Levine et al.	2021	10.5
Communications	Ethiopia	Childhood immunizations	Mekonnen et al.	2021	11.7
Communications	Ethiopia	Penta-3	Mekonnen et al.	2021	8.9
Communications	Ethiopia	Measles	Mekonnen et al.	2021	12.2
Communications	USA	Influenza	Milkman et al.	2021	2.1
Communications	China	Varicella	Qin et al.	2021	10.8
Communications	China	Varicella	Qin et al.	2021	11.7
Communications	USA	Childhood immunizations	Wagner et al.	2021	0
Communications	USA	HPV	Bastani et al.	2022	0
Communications	USA	COVID-19	Jacobson et al.	2022	0

Intervention Type	Country of Study	Vaccine(s) Studied	Author	Publication Year	Effect Size
Communications	USA	COVID-19	Jacobson et al.	2022	0
Communications	USA	COVID-19	Mehta et al.	2022	0
Communications	USA	COVID-19	Mehta et al.	2022	0
Communications	USA	Influenza	Milkman et al.	2022	2
Communications	Australia	Childhood immunizations (7 months)	O'Grady et al.	2022	15.9
Communications	USA	COVID-19	Patel et al.	2022	4.9
Communications	USA	COVID-19	Rabb et al.	2022	0
Communications	Finland	Influenza	Sääksvuori et al.	2022	6.4
Communications	Italy	COVID-19	Tentori et al.	2022	3.2
Communications	USA	Herpes zoster	Gatwood et al.	2023	3
Community Actions	Pakistan	Oral Polio Vaccine	Habib et al.	2017	9
Community Actions	Pakistan	Oral Polio Vaccine	Habib et al.	2017	7
Community Actions	India	Children fully immunised against six VPDs	Sengupta et al.	2017	17.3
Community Actions	India	DTP3	Nagar et al.	2018	0
Community Actions	India	DTP3	Nagar et al.	2018	0
Community Actions	Pakistan	Measles	Siddiqi et al.	2020	0
Community Actions	Pakistan	Pentavalent 3	Siddiqi et al.	2020	0
Community Actions	Sierra Leone	Childhood immunizations	Karing	2021	14

Intervention Type	Country of Study	Vaccine(s) Studied	Author	Publication Year	Effect Size
Community Actions	Cameroon	Specific Immunization Completeness	Ateudjieu et al.	2022	0
Community Actions	Cameroon	General Immunization Completeness	Ateudjieu et al.	2022	0
Community Actions	USA	COVID-19	Lieu et al.	2022	2.3
Community Actions	USA	COVID-19	Lieu et al.	2022	1.4
Education	Australia	Hepatitis B	Skinner et al.	2000	0
Education	USA	Childhood immunizations	Ashkar et al.	2003	0
Education	USA	Influenza	Kimura et al.	2007	0
Education	USA	Influenza	Kimura et al.	2007	19
Education	USA	Influenza	Kimura et al.	2007	26
Education	India	Tetanus	Pandey et al.	2007	24
Education	India	General Infant Vaccinations	Pandey et al.	2007	26
Education	Pakistan	Measles	Andersson et al.	2009	20
Education	Pakistan	DPT3 Full Course	Andersson et al.	2009	28.5
Education	Japan	Influenza	Usami et al.	2009	16.7
Education	Pakistan	DPT/Hepatitis B	Owais et al.	2011	20.4
Education	Pakistan	DPT3	Usman et al.	2011	22
Education	Pakistan	DPT3	Usman et al.	2011	27
Education	Pakistan	DPT3	Usman et al.	2011	28
Education	USA	HPV	Hopfer	2012	6
Education	USA	HPV	Hopfer	2012	10
Education	USA	HPV	Hopfer	2012	-5.8
Education	Spain	Influenza	Roca et al.	2012	5.4

Intervention Type	Country of Study	Vaccine(s) Studied	Author	Publication Year	Effect Size
Education	USA	Influenza	Frew et al.	2014	0
Education	USA	Influenza	Frew et al.	2014	0
Education	USA	HPV	Bennett et al.	2015	0
Education	USA	HPV initiation	DiClemente et al.	2015	0
Education	USA	Influenza	Goodman et al.	2015	0
Education	USA	HPV	Tiro et al.	2015	0
Education	USA	Influenza	Yoo et al.	2015	12
Education	Sweden	HPV	Grandahl et al.	2016	6.5
Education	USA	HPV initiation	Joseph et al.	2016	0
Education	USA	HPV completion	Joseph et al.	2016	0
Education	Hong Kong	Influenza	Wong et al.	2016	11.1
Education	China	Full immunisation (mothers)	Hu et al.	2017	7.1
Education	Hong Kong	Influenza	Leung et al.	2017	8.6
Education	France	Influenza	Berkhout et al.	2018	0
Education	Australia	Influenza	Borg et al.	2018	1.5
Education	USA	HPV series completion	Dempsey et al.	2018	4.4
Education	USA	HPV series intitiation	Dempsey et al.	2018	9.5
Education	India	DPT3	Powell-Jackson et al.	2018	15
Education	USA	Zoster	Whitaker et al.	2018	4.2
Education	USA	Pertussis	Whitaker et al.	2018	0
Education	USA	Influenza	Whitaker et al.	2018	0
Education	USA	Tetanus	Whitaker et al.	2018	0
Education	USA	Pneumococcal	Whitaker et al.	2018	0
Education	Hong Kong	Influenza	Yeung et al.	2018	26

Intervention Type	Country of Study	Vaccine(s) Studied	Author	Publication Year	Effect Size
Education	USA	HPV	Dixon et al.	2019	14.7
Education	Singapore	Influenza	Ho et al.	2019	.9
Education	Singapore	Pneumococcal	Ho et al.	2019	2
Education	USA	TDAP; MenACWY; HPV	Underwood et al.	2019	10.6
Education	USA	TDAP; MenACWY; HPV	Underwood et al.	2019	6.8
Education	USA	HPV	Kim et al.	2020	8.5
Education	India	Full immunisation (children)	Pramanik et al.	2020	0
Education	USA	Childhood immunizations	Kappes et al.	2021	0
Education	Nigeria	Pentavalent 2	Oyo-Ita et al.	2021	9
Education	Nigeria	Measles	Oyo-Ita et al.	2021	17
Education	Nigeria	Pentavalent 3	Oyo-Ita et al.	2021	5
Education	Nigeria	Pentavalent 1	Oyo-Ita et al.	2021	17
Education	USA	HPV	Santa Maria et al.	2021	9.7
Education	China	Influenza	Jiang et al.	2022	6.9
Education	USA	Influenza	Marshall et al.	2022	2.06
Education	China	HPV	Si et al.	2022	0
Healthcare Improvement	Netherlands	Influenza	Hak et al.	2000	7
Healthcare Improvement	UK	Pneumococcal (diabetes patients)	Siriwardena et al.	2002	8.7
Healthcare Improvement	UK	Pneumococcal (CHD patients)	Siriwardena et al.	2002	8.3
Healthcare Improvement	UK	Pneumococcal (splenectomy patients)	Siriwardena et al.	2002	0
Healthcare Improvement	USA	Childhood immunizations	Hambidge et al.	2004	6

Intervention Type	Country of Study	Vaccine(s) Studied	Author	Publication Year	Effect Size
Healthcare Improvement	India	Full immunisation (children)	Banerjee et al.	2010	12
Healthcare Improvement	USA	Childhood immunizations (12–23 months)	Boom et al.	2010	0
Healthcare Improvement	USA	Influenza	Nowalk et al.	2010	0
Healthcare Improvement	USA	Influenza	Nowalk et al.	2010	0
Healthcare Improvement	USA	Influenza	Bond et al.	2011	5.7
Healthcare Improvement	Ethiopia	Measles	Carnell et al.	2014	8.4
Healthcare Improvement	Ethiopia	DPT3	Carnell et al.	2014	14.4
Healthcare Improvement	USA	Influenza	Nowalk et al.	2014	5.7
Healthcare Improvement	Guatemala	Complete childhood immunisation	Busso et al.	2015	2.2
Healthcare Improvement	USA	All necessary vaccines	Fu et al.	2016	0
Healthcare Improvement	USA	HPV completion	Zimmerman et al.	2017	0
Healthcare Improvement	USA	HPV initiation	Zimmerman et al.	2017	2.9
Healthcare Improvement	France	Influenza	Borgey et al.	2019	10.8
Healthcare Improvement	USA	Influenza; TDAP; HPV	O'Leary et al.	2019	0
Healthcare Improvement	USA	HPV	Szilagyi et al.	2021	2.4

Intervention Type	Country of Study	Vaccine(s) Studied	Author	Publication Year	Effect Size
Healthcare Improvement	USA	Combination 10	Williams et al.	2021	0
Healthcare Improvement	USA	COVID-19	Berry et al.	2022	0
Healthcare Improvement	USA	HPV initiation	Gilkey et al.	2022	3.8
Healthcare Improvement	USA	HPV initiation	Gilkey et al.	2022	1.5
Healthcare Improvement	USA	Influenza (19–64 yrs)	Heaton et al.	2022	0
Healthcare Improvement	USA	Influenza ($\geq$ 65 yrs)	Heaton et al.	2022	0
Incentives	USA	MMR; Poliovirus; Diphtheria; Tetanus Toxoids and Pertussis; Haemophilus influenzae type b; Hepatitis B.	Kerpelman et al.	2000	11.8
Incentives	USA	Any vaccination	Fairbrother et al.	2001	5.9
Incentives	USA	Any vaccination	Fairbrother et al.	2001	7.4
Incentives	USA	Influenza	Doratotaj et al.	2008	0
Incentives	USA	Influenza	Doratotaj et al.	2008	0
Incentives	India	Full immunisation (children)	Banerjee et al.	2010	33
Incentives	Australia	Hepatitis B	Topp et al.	2013	21
Incentives	UK	Hepatitis B	Weaver et al.	2014	40
Incentives	UK	Hepatitis B	Weaver et al.	2014	36
Incentives	UK	HPV initiation	Mantzari et al.	2015	8.8
Incentives	USA	All necessary vaccines	Fu et al.	2016	0
Incentives	Nigeria	Tetanus toxoid	Sato et al.	2020	30.7

Intervention Type	Country of Study	Vaccine(s) Studied	Author	Publication Year	Effect Size
Incentives	Nigeria	Tetanus toxoid	Sato et al.	2020	20.9
Incentives	Singapore	Influenza	Yue et al.	2020	4.7
Incentives	Singapore	Influenza	Yue et al.	2020	3
Incentives	Sweden	COVID-19	Campos-Mercade et al.	2021	4.2
Incentives	USA	COVID-19	Chang et al.	2021	1
Incentives	Ghana	Polio; BCG	Levine et al.	2021	49.5
Incentives	USA	COVID-19	Jacobson et al.	2022	0
Incentives	USA	COVID-19	Lang et al.	2022	.7
Incentives	USA	COVID-19	Milkman et al.	2022	.06

Notes: This table reports the intervention types, locations, vaccines administered, and effect sizes in percentage points for RCTs testing interventions used to increase the uptake of vaccines. The reported studies were conducted across a variety of countries, use various intervention types, and different types of vaccines are included. These studies were found through searching databases, reviewing prior systematic reviews, and reviewing the references of related studies. Across these studies, the mean effect size of the reported change in vaccination rate is 6.81 percentage points (SD 9.05). FIC here refers to "fully immunized child", or a child who has received one dose of BCG vaccine, three doses of DPT vaccine, three doses of oral polio vaccine, and one dose of the measles vaccine.

Bibliography of Literature Review Studies

[0] Anna-Corinna Kulle, Stefanie Schumacher, and Frauke von Bieberstein. "Mobile vaccination units substantially increase COVID-19 vaccinations: evidence from a randomized controlled trial". In: *Journal of public health (Oxford, England)* (Nov. 2023), fdad213. ISSN: 1741-3842. DOI: 10.1093/pubmed/fdad213. URL: https://doi.org/10.1093/pubmed/fdad213.

[1] Zvi Howard Abramson et al. "Randomized Trial of a Program to Increase Staff Influenza Vaccination in Primary Care Clinics." In: *Annals of family medicine* 8.4 (2010 Jul-Aug), pp. 293–298. ISSN: 1544-1717 1544-1709. DOI: 10.1370/afm.1132. pmid: 20644183.

[2] Faruque Ahmed et al. "Effect of the Frequency of Delivery of Reminders and an Influenza Tool Kit on Increasing Influenza Vaccination Rates among Adults with High-Risk Conditions." In: *The American journal of managed care* 10.10 (Oct. 2004), pp. 698–702. ISSN: 1088-0224. pmid: 15521161.

[3] Neil Andersson et al. "Evidence-Based Discussion Increases Childhood Vaccination Uptake: A Randomised Cluster Controlled Trial of Knowledge Translation in Pakistan". In: *BMC International Health and Human Rights* 9.S1 (Oct. 2009), S8. ISSN: 1472-698X. DOI: 10.1186/1472-698X-9-S1-S8. URL: https://bmcinthealthhumrights.biomedcentral.com/articles/10.1186/1472-698X-9-S1-S8 (visited on 01/16/2023).

[4] Antony J. Arthur et al. "Improving Uptake of Influenza Vaccination among Older People: A Randomised Controlled Trial." In: *The British journal of general practice : the journal of the Royal College of General Practitioners* 52.482 (Sept. 2002), pp. 717–8, 720–722. ISSN: 0960-1643. pmid: 12236274.

[5] Susan H. Ashkar et al. "The Effectiveness of Assessment and Referral on Immunization Coverage in the Special Supplemental Nutrition Program for Women, Infants, and Children." In: *Archives of pediatrics & adolescent medicine* 157.5 (May 2003), pp. 456–462. ISSN: 1072-4710. DOI: 10.1001/archpedi.157.5.456. pmid: 12742881.

[6] Jérôme Ateudjieu et al. "Tracking Demographic Movements and Immunization Status to Improve Children's Access to Immunization: Field-Based Randomized Controlled Trial." In: *JMIR public health and surveillance* 8.3 (Mar. 1, 2022), e32213. ISSN: 2369-2960. DOI: 10.2196/32213. pmid: 35230249.

[7] Abhijit Vinayak Banerjee et al. "Improving Immunisation Coverage in Rural India: Clustered Randomised Controlled Evaluation of Immunisation Campaigns with and without Incentives." In: *BMJ (Clinical research ed.)* 340 (May 17, 2010), p. c2220. ISSN: 1756-1833 0959-8138. DOI: 10.1136/bmj.c2220. pmid: 20478960.

[8] Donewell Bangure et al. "Effectiveness of Short Message Services Reminder on Childhood Immunization Programme in Kadoma, Zimbabwe - a Randomized Controlled Trial, 2013." In: *BMC public health* 15 (Feb. 12, 2015), p. 137. ISSN: 1471-2458. DOI: 10.1186/s12889-015-1470-6. pmid: 25885862.

[9] Adriaan Barbaroux et al. "Nudging Health Care Workers towards a Flu Shot: Reminders Are Accepted but Not Necessarily Effective. A Randomized Controlled Study among Residents in General Practice in France." In: *Family practice* 38.4 (July 28, 2021), pp. 410–415. ISSN: 1460-2229 0263-2136. DOI: 10.1093/fampra/cmab001. pmid: 33506858.

[10] Roshan Bastani et al. "Increasing HPV Vaccination among Low-Income, Ethnic Minority Adolescents: Effects of a Multicomponent System Intervention through a County Health Department Hotline." In: *Cancer epidemiology, biomarkers & prevention : a publication of the American Association for Cancer Research, cosponsored by the American Society of Preventive Oncology* 31.1 (Jan. 2022), pp. 175–182. ISSN: 1538-7755 1055-9965. DOI: 10.1158/1055-9965.EPI-20-1578. pmid: 34649960.

[11] Alaina T. Bennett et al. "Human Papillomavirus Vaccine Uptake After a Tailored, Online Educational Intervention for Female University Students: A Randomized Controlled Trial." In: *Journal of women's health (2002)* 24.11 (Nov. 2015), pp. 950–957. ISSN: 1931-843X 1540-9996. DOI: 10.1089/jwh.2015.5251. pmid: 26488269.

[12] Christophe Berkhout et al. "Randomized Controlled Trial on Promoting Influenza Vaccination in General Practice Waiting Rooms." In: *PloS one* 13.2 (2018), e0192155. ISSN: 1932-6203. DOI: 10.1371/journal.pone.0192155. pmid: 29425226.

[13] Marie-Pier Bernard-Genest, Jessica Ruel-Laliberté, and Korine Lapointe-Milot. "Effect of Educative Reminder Telephone Calls on Human Papillomavirus Immunization Rate: A Randomized Controlled Trial." In: *Women's health (London, England)* 17 (2021 Jan-Dec), p. 17455065211003821. ISSN: 1745-5065 1745-5057. DOI: 10.1177/17455065211003821. pmid: 33779417.

[14] Sarah D. Berry et al. "Evaluating the Findings of the IMPACT-C Randomized Clinical Trial to Improve COVID-19 Vaccine Coverage in Skilled Nursing Facilities." In: *JAMA internal medicine* 182.3 (Mar. 1, 2022), pp. 324–331. ISSN: 2168-6114 2168-6106. DOI: 10.1001/jamainternmed.2021.8067. pmid: 35099523.

[15] T. Christopher Bond et al. "A Group-Randomized Evaluation of a Quality Improvement Intervention to Improve Influenza Vaccination Rates in Dialysis Centers." In: *American journal of kidney diseases : the official journal of the National Kidney Foundation* 57.2 (Feb. 2011), pp. 283–290. ISSN: 1523-6838 0272-6386. DOI: 10.1053/j.ajkd.2010.09.019. pmid: 21146267.

[16] Julie A. Boom et al. "Utilizing Peer Academic Detailing to Improve Childhood Immunization Coverage Levels." In: *Health promotion practice* 11.3 (May 2010), pp. 377–386. ISSN: 1524-8399. DOI: 10.1177/1524839908321487. pmid: 18955545.

[17] Kim Borg et al. "Communication-Based Interventions for Increasing Influenza Vaccination Rates among Aboriginal Children: A Randomised Controlled Trial." In: *Vaccine* 36.45 (Oct. 29, 2018), pp. 6790–6795. ISSN: 1873-2518 0264-410X. DOI: 10.1016/j.vaccine.2018.09.020. pmid: 30279091.

[18] France Borgey et al. "Effectiveness of an Intervention Campaign on Influenza Vaccination of Professionals in Nursing Homes: A Cluster-Randomized Controlled Trial." In: *Vaccine* 37.10 (Feb. 28, 2019), pp. 1260–1265. ISSN: 1873-2518 0264-410X. DOI: 10.1016/j.vaccine.2019.01.066. pmid: 30738645.

[19] Matias Busso, Julian Cristia, and Sarah Humpage. "Did You Get Your Shots? Experimental Evidence on the Role of Reminders." In: *Journal of health economics* 44 (Dec. 2015), pp. 226–237. ISSN: 1879-1646 0167-6296. DOI: 10.1016/j.jhealeco.2015.08.005. pmid: 26519909.

[20] Pol Campos-Mercade et al. "Monetary Incentives Increase COVID-19 Vaccinations." In: *Science (New York, N.Y.)* 374.6569 (Nov. 12, 2021), pp. 879–882. ISSN: 1095-9203 0036-8075. DOI: 10.1126/science.abm0475. pmid: 34618594.

[21] Mary A. Carnell et al. "Effectiveness of Scaling up the 'three Pillars' Approach to Accelerating MDG 4 Progress in Ethiopia". In: *Journal of Health, Population, and Nutrition* 32.4 (Dec. 2014), pp. 549–563. ISSN: 1606-0997. pmid: 25895187.

[22] Tom Chang et al. *Financial Incentives and Other Nudges Do Not Increase COVID-19 Vaccinations among the Vaccine Hesitant*. w29403. Cambridge, MA: National Bureau of Economic Research, Oct. 2021, w29403. DOI: 10.3386/w29403. URL: http://www.nber.org/papers/w29403.pdf (visited on 01/16/2023).

[23] Chun Chao et al. "A Randomized Intervention of Reminder Letter for Human Papillomavirus Vaccine Series Completion." In: *The Journal of adolescent health : official publication of the Society for Adolescent Medicine* 56.1 (Jan. 2015), pp. 85–90. ISSN: 1879-1972 1054-139X. DOI: 10.1016/j.jadohealth.2014.08.014. pmid: 25438965.

[24] Gretchen B. Chapman et al. "Opting In vs Opting Out of Influenza Vaccination". In: *JAMA* 304.1 (July 7, 2010), p. 43. ISSN: 0098-7484. DOI: 10.1001/jama.2010.892. URL: http://jama.jamanetwork.com/article.aspx?doi=10.1001/jama.2010.892 (visited on 01/16/2023).

[25] Li Chen et al. "Effectiveness of a Smartphone App on Improving Immunization of Children in Rural Sichuan Province, China: A Cluster Randomized Controlled Trial." In: *BMC public health* 16.1 (Aug. 31, 2016), p. 909. ISSN: 1471-2458. DOI: 10.1186/s12889-016-3549-0. pmid: 27581655.

[26] Nuole Chen et al. "The Effect of Postcard Reminders on Vaccinations among the Elderly: A Block-Randomized Experiment". In: *Behavioural Public Policy* (July 23, 2020), pp. 1–26. ISSN: 2398-063X, 2398-0648. DOI: 10.1017/bpp.2020.34. URL: https://www.cambridge.org/core/product/identifier/S2398063X20000342/type/journal_article (visited on 01/16/2023).

[27] Hengchen Dai et al. "Behavioural Nudges Increase COVID-19 Vaccinations." In: *Nature* 597.7876 (Sept. 2021), pp. 404–409. ISSN: 1476-4687 0028-0836. DOI: 10.1038/s41586-021-03843-2. pmid: 34340242.

[28] Amanda F. Dempsey et al. "A Randomized, Controlled, Pragmatic Trial of an iPad-based, Tailored Messaging Intervention to Increase Human Papillomavirus Vaccination among Latinos." In: *Human vaccines & immunotherapeutics* 15.7-8 (2019), pp. 1577–1584. ISSN: 2164-554X 2164-5515. DOI: 10.1080/21645515.2018.1559685. pmid: 30689494.

[29] Amanda F. Dempsey et al. "Effect of a Health Care Professional Communication Training Intervention on Adolescent Human Papillomavirus Vaccination: A Cluster Randomized Clinical Trial." In: *JAMA pediatrics* 172.5 (May 7, 2018), e180016. ISSN: 2168-6211 2168-6203. DOI: 10.1001/jamapediatrics.2018.0016. pmid: 29507952.

[30] Ralph J. DiClemente et al. "Overcoming Barriers to HPV Vaccination: A Randomized Clinical Trial of a Culturally-Tailored, Media Intervention among African American Girls." In: *Human vaccines & immunotherapeutics* 11.12 (2015), pp. 2883–2894. ISSN: 2164-554X 2164-5515. DOI: 10.1080/21645515.2015.1070996. pmid: 26378650.

[31] E. F. Dini, R. W. Linkins, and J. Sigafoos. "The Impact of Computer-Generated Messages on Childhood Immunization Coverage." In: *American journal of preventive medicine* 18.2 (Feb. 2000), pp. 132–139. ISSN: 0749-3797. DOI: 10.1016/s0749-3797(99)00086-0. pmid: 10698243.

[32] Brian E. Dixon et al. "An Educational Intervention to Improve HPV Vaccination: A Cluster Randomized Trial." In: *Pediatrics* 143.1 (Jan. 2019), e20181457. ISSN: 1098-4275 0031-4005. DOI: 10.1542/peds.2018-1457. pmid: 30530637.

[33] Kevin J. Dombkowski et al. "Age-Specific Strategies for Immunization Reminders and Recalls: A Registry-Based Randomized Trial." In: *American journal of preventive medicine* 47.1 (July 2014), pp. 1–8. ISSN: 1873-2607 0749-3797. DOI: 10.1016/j.amepre.2014.02.009. pmid: 24750973.

[34] Kevin J. Dombkowski et al. "Seasonal Influenza Vaccination Reminders for Children with High-Risk Conditions: A Registry-Based Randomized Trial." In: *American journal of preventive medicine* 42.1 (Jan. 2012), pp. 71–75. ISSN: 1873-2607 0749-3797. DOI: 10.1016/j.amepre.2011.09.028. pmid: 22176850.

[35] Shirin Doratotaj, Michael L. Macknin, and Sarah Worley. "A Novel Approach to Improve Influenza Vaccination Rates among Health Care Professionals: A Prospective Randomized Controlled Trial." In: *American journal of infection control* 36.4 (May 2008), pp. 301–303. ISSN: 1527-3296 0196-6553. DOI: 10.1016/j.ajic.2007.10.019. pmid: 18455052.

[36] G. Fairbrother et al. "Impact of Financial Incentives on Documented Immunization Rates in the Inner City: Results of a Randomized Controlled Trial." In: *Ambulatory pediatrics : the official journal of the Ambulatory Pediatric Association* 1.4 (2001 Jul-Aug), pp. 206–212. ISSN: 1530-1567. DOI: 10.1367/1539-4409(2001)001<0206:iofiod>2.0.co;2. pmid: 11888402.

[37] Paula M. Frew et al. "Socioecological and Message Framing Factors Influencing Maternal Influenza Immunization among Minority Women." In: *Vaccine* 32.15 (Mar. 26, 2014), pp. 1736–1744. ISSN: 1873-2518 0264-410X. DOI: 10.1016/j.vaccine.2014.01.030. pmid: 24486366.

[38] Linda Y. Fu et al. "Strategies for Improving Vaccine Delivery: A Cluster-Randomized Trial." In: *Pediatrics* 137.6 (June 2016), e20154603. ISSN: 1098-4275 0031-4005. DOI: 10.1542/peds.2015-4603. pmid: 27244859.

[39] Justin Gatwood et al. "Impact of Patient and Provider Nudges on Addressing Herpes Zoster Vaccine Series Completion." In: *Vaccine* 41.3 (Jan. 16, 2023), pp. 778–786. ISSN: 1873-2518 0264-410X. DOI: 10.1016/j.vaccine.2022.12.016. pmid: 36526504.

[40] Mary A. Gerend et al. "Evaluation of a Text Messaging-Based Human Papillomavirus Vaccination Intervention for Young Sexual Minority Men: Results from a Pilot Randomized Controlled Trial." In: *Annals of behavioral medicine : a publication of the Society of Behavioral Medicine* 55.4 (Apr. 7, 2021), pp. 321–332. ISSN: 1532-4796 0883-6612. DOI: 10.1093/abm/kaaa056. pmid: 32914838.

[41] Alexandra S. Ghadieh et al. "The Effect of Various Types of Patients' Reminders on the Uptake of Pneumococcal Vaccine in Adults: A Randomized Controlled Trial." In: *Vaccine* 33.43 (Oct. 26, 2015), pp. 5868–5872. ISSN: 1873-2518 0264-410X. DOI: 10.1016/j.vaccine.2015.07.050. pmid: 26232345.

[42] Melissa B. Gilkey et al. "Coaching and Communication Training for HPV Vaccination: A Cluster Randomized Trial." In: *Pediatrics* 150.2 (Aug. 1, 2022). ISSN: 1098-4275 0031-4005. DOI: 10.1542/peds.2021-052351. pmid: 35818840.

[43] Jason M. Glanz et al. "Web-Based Social Media Intervention to Increase Vaccine Acceptance: A Randomized Controlled Trial." In: *Pediatrics* 140.6 (Dec. 2017). ISSN: 1098-4275 0031-4005. DOI: 10.1542/peds.2017-1117. pmid: 29109107.

[44] Kenneth Goodman et al. “Impact of Video Education on Influenza Vaccination in Pregnancy.” In: *The Journal of reproductive medicine* 60.11-12 (2015 Nov-Dec), pp. 471–479. ISSN: 0024-7758. pmid: 26775454.

[45] Maria Grandahl et al. “School-Based Intervention for the Prevention of HPV among Adolescents: A Cluster Randomised Controlled Study.” In: *BMJ open* 6.1 (Jan. 27, 2016), e009875. ISSN: 2044-6055. DOI: 10.1136/bmjopen-2015-009875. pmid: 26817639.

[46] Dennis Gurfinkel et al. “Centralized Reminder/Recall for Human Papillomavirus Vaccination: Findings From Two States-A Randomized Clinical Trial.” In: *The Journal of adolescent health : official publication of the Society for Adolescent Medicine* 69.4 (Oct. 2021), pp. 579–587. ISSN: 1879-1972 1054-139X. DOI: 10.1016/j.jadohealth.2021.02.023. pmid: 33846054.

[47] Muhammad Atif Habib et al. “Community Engagement and Integrated Health and Polio Immunisation Campaigns in Conflict-Affected Areas of Pakistan: A Cluster Randomised Controlled Trial.” In: *The Lancet. Global health* 5.6 (June 2017), e593–e603. ISSN: 2214-109X. DOI: 10.1016/S2214-109X(17)30184-5. pmid: 28495264.

[48] E. Hak et al. “Effectiveness of a Co-Ordinated Nation-Wide Programme to Improve Influenza Immunisation Rates in The Netherlands.” In: *Scandinavian journal of primary health care* 18.4 (Dec. 2000), pp. 237–241. ISSN: 0281-3432. DOI: 10.1080/028134300448814. pmid: 11205093.

[49] Simon J. Hambidge et al. “Strategies to Improve Immunization Rates and Well-Child Care in a Disadvantaged Population: A Cluster Randomized Controlled Trial.” In: *Archives of pediatrics & adolescent medicine* 158.2 (Feb. 2004), pp. 162–169. ISSN: 1072-4710. DOI: 10.1001/archpedi.158.2.162. pmid: 14757608.

[50] Pamela C. Heaton et al. “The Impact of Community Pharmacy Utilization of Immunization Information Systems on Vaccination Rates: Results of a Clustered Randomized Controlled Trial.” In: *Journal of the American Pharmacists Association : JAPhA* 62.1 (2022 Jan-Feb), 95–103.e2. ISSN: 1544-3450 1086-5802. DOI: 10.1016/j.japh.2021.09.010. pmid: 34764037.

[51] Emily Herrett et al. “Text Messaging Reminders for Influenza Vaccine in Primary Care: A Cluster Randomised Controlled Trial (TXT4FLUJAB).” In: *BMJ open* 6.2 (Feb. 19, 2016), e010069. ISSN: 2044-6055. DOI: 10.1136/bmjopen-2015-010069. pmid: 26895984.

[52] Rick Hess. “Impact of Automated Telephone Messaging on Zoster Vaccination Rates in Community Pharmacies.” In: *Journal of the American Pharmacists Association : JAPhA* 53.2 (2013 Mar-Apr), pp. 182–187. ISSN: 1544-3450 1086-5802. DOI: 10.1331/JAPhA.2013.12222. pmid: 23571626.

[53] Hanley J. Ho et al. "Increasing Influenza and Pneumococcal Vaccination Uptake in Seniors Using Point-of-Care Informational Interventions in Primary Care in Singapore: A Pragmatic, Cluster-Randomized Crossover Trial." In: *American journal of public health* 109.12 (Dec. 2019), pp. 1776–1783. ISSN: 1541-0048 0090-0036. DOI: 10.2105/AJPH.2019.305328. pmid: 31622142.

[54] Annika M. Hofstetter et al. "Impacting Delayed Pediatric Influenza Vaccination: A Randomized Controlled Trial of Text Message Reminders." In: *American journal of preventive medicine* 48.4 (Apr. 2015), pp. 392–401. ISSN: 1873-2607 0749-3797. DOI: 10.1016/j.amepre.2014.10.023. pmid: 25812465.

[55] Suellen Hopfer. "Effects of a Narrative HPV Vaccination Intervention Aimed at Reaching College Women: A Randomized Controlled Trial." In: *Prevention science : the official journal of the Society for Prevention Research* 13.2 (Apr. 2012), pp. 173–182. ISSN: 1573-6695 1389-4986. DOI: 10.1007/s11121-011-0254-1. pmid: 21993613.

[56] Yu Hu et al. "Prenatal Vaccination Education Intervention Improves Both the Mothers' Knowledge and Children's Vaccination Coverage: Evidence from Randomized Controlled Trial from Eastern China." In: *Human vaccines & immunotherapeutics* 13.6 (June 3, 2017), pp. 1–8. ISSN: 2164-554X 2164-5515. DOI: 10.1080/21645515.2017.1285476. pmid: 28319453.

[57] Sharon G. Humiston et al. "Increasing Inner-City Adult Influenza Vaccination Rates: A Randomized Controlled Trial." In: *Public health reports (Washington, D.C. : 1974)* 126 Suppl 2 (Suppl 2 2011 Jul-Aug), pp. 39–47. ISSN: 0033-3549 1468-2877. DOI: 10.1177/00333549111260S206. pmid: 21812168.

[58] Laura P. Hurley et al. "RCT of Centralized Vaccine Reminder/Recall for Adults." In: *American journal of preventive medicine* 55.2 (Aug. 2018), pp. 231–239. ISSN: 1873-2607 0749-3797. DOI: 10.1016/j.amepre.2018.04.022. pmid: 29910118.

[59] Annabel Ibáñez-Jiménez et al. "[Randomized clinical trial on the effectiveness of a postal reminder to increase tetanus-diphtheria vaccination coverage in the young adult population]." In: *Enfermeria clinica* 17.4 (2007 Jul-Aug), pp. 171–176. ISSN: 1130-8621. DOI: 10.1016/s1130-8621(07)71793-x. pmid: 17915119.

[60] Mireille Jacobson et al. "Can Financial Incentives and Other Nudges Increase COVID-19 Vaccinations among the Vaccine Hesitant? A Randomized Trial." In: *Vaccine* 40.43 (Oct. 12, 2022), pp. 6235–6242. ISSN: 1873-2518 0264-410X. DOI: 10.1016/j.vaccine.2022.08.060. pmid: 36137900.

[61] Minghuan Jiang et al. "Impact of Video-Led Educational Intervention on Uptake of Influenza Vaccine among the Elderly in Western China: A Community-Based Randomized Controlled Trial." In: *BMC public health* 22.1 (June 6, 2022), p. 1128. ISSN: 1471-2458. DOI: 10.1186/s12889-022-13536-8. pmid: 35668438.

[62] Natalie Pierre Joseph et al. "Brief Client-Centered Motivational and Behavioral Intervention to Promote HPV Vaccination in a Hard-to-Reach Population: A Pilot Randomized Controlled Trial." In: *Clinical pediatrics* 55.9 (Aug. 2016), pp. 851–859. ISSN: 1938-2707 0009-9228. DOI: 10.1177/0009922815616244. pmid: 26968631.

[63] E. Wangeci Kagucia et al. "Impact of Mobile Phone Delivered Reminders and Unconditional Incentives on Measles-Containing Vaccine Timeliness and Coverage: A Randomised Controlled Trial in Western Kenya." In: *BMJ global health* 6.1 (Jan. 2021). ISSN: 2059-7908. DOI: 10.1136/bmjgh-2020-003357. pmid: 33509838.

[64] Heather Kappes et al. "Lessons for Covid-19 Vaccination from Eight Federal Government Direct Communication Evaluations". In: *SSRN Electronic Journal* (2021). ISSN: 1556-5068. DOI: 10.2139/ssrn.3967610. URL: https://www.ssrn.com/abstract=3967610 (visited on 01/16/2023).

[65] Anne Karing. "Social signaling and childhood immunization: A field experiment in Sierra Leone". In: *University of California, Berkeley* 2 (2018).

[66] Yoshito Kawakatsu et al. "Cost-Effectiveness of SMS Appointment Reminders in Increasing Vaccination Uptake in Lagos, Nigeria: A Multi-Centered Randomized Controlled Trial." In: *Vaccine* 38.42 (Sept. 29, 2020), pp. 6600–6608. ISSN: 1873-2518 0264-410X. DOI: 10.1016/j.vaccine.2020.07.075. pmid: 32788139.

[67] Allison Kempe et al. "Centralized Reminder/Recall to Increase Immunization Rates in Young Children: How Much Bang for the Buck?" In: *Academic pediatrics* 17.3 (Apr. 2017), pp. 330–338. ISSN: 1876-2867 1876-2859. DOI: 10.1016/j.acap.2016.11.016. pmid: 27913163.

[68] L. C. Kerpelman, D. B. Connell, and W. J. Gunn. "Effect of a Monetary Sanction on Immunization Rates of Recipients of Aid to Families with Dependent Children." In: *JAMA* 284.1 (July 5, 2000), pp. 53–59. ISSN: 0098-7484. DOI: 10.1001/jama.284.1.53. pmid: 10872013.

[69] Minjin Kim et al. "A Storytelling Intervention in a Mobile, Web-Based Platform: A Pilot Randomized Controlled Trial to Evaluate the Preliminary Effectiveness to Promote Human Papillomavirus Vaccination in Korean American College Women." In: *Health education & behavior : the official publication of the Society for Public Health Education* 47.2 (Apr. 2020), pp. 258–263. ISSN: 1552-6127 1090-1981. DOI: 10.1177/1090198119894589. pmid: 31958991.

[70] Akiko C. Kimura et al. "The Effectiveness of Vaccine Day and Educational Interventions on Influenza Vaccine Coverage among Health Care Workers at Long-Term Care Facilities." In: *American journal of public health* 97.4 (Apr. 2007), pp. 684–690. ISSN: 1541-0048 0090-0036. DOI: 10.2105/AJPH.2005.082073. pmid: 17329659.

[71] J. W. Krieger et al. "Increasing Influenza and Pneumococcal Immunization Rates: A Randomized Controlled Study of a Senior Center-Based Intervention." In: *American journal of preventive medicine* 18.2 (Feb. 2000), pp. 123–131. ISSN: 0749-3797. DOI: 10.1016/s0749-3797(99)00134-8. pmid: 10698242.

[72] David Lang, Lief Esbenshade, and Robb Willer. "Did Ohio's Vaccine Lottery Increase Vaccination Rates? A Pre-Registered, Synthetic Control Study". In: *Journal of Experimental Political Science* (Feb. 7, 2022), pp. 1–19. ISSN: 2052-2630, 2052-2649. DOI: 10.1017/XPS.2021.32. URL: https://www.cambridge.org/core/product/identifier/S2052263021000324/type/journal_article (visited on 01/16/2023).

[73] Wei-Nchih Lee et al. "Large-Scale Influenza Vaccination Promotion on a Mobile App Platform: A Randomized Controlled Trial." In: *Vaccine* 38.18 (Apr. 16, 2020), pp. 3508–3514. ISSN: 1873-2518 0264-410X. DOI: 10.1016/j.vaccine.2019.11.053. pmid: 31787410.

[74] Birthe A. Lehmann et al. "Changing the Default to Promote Influenza Vaccination among Health Care Workers." In: *Vaccine* 34.11 (Mar. 8, 2016), pp. 1389–1392. ISSN: 1873-2518 0264-410X. DOI: 10.1016/j.vaccine.2016.01.046. pmid: 26851734.

[75] Ka Chun Leung et al. "Impact of Patient Education on Influenza Vaccine Uptake among Community-Dwelling Elderly: A Randomized Controlled Trial." In: *Health education research* 32.5 (Oct. 1, 2017), pp. 455–464. ISSN: 1465-3648 0268-1153. DOI: 10.1093/her/cyx053. pmid: 28931164.

[76] Gillian Levine et al. "Mobile Nudges and Financial Incentives to Improve Coverage of Timely Neonatal Vaccination in Rural Areas (GEVaP Trial): A 3-Armed Cluster Randomized Controlled Trial in Northern Ghana." In: *PloS one* 16.5 (2021), e0247485. ISSN: 1932-6203. DOI: 10.1371/journal.pone.0247485. pmid: 34010312.

[77] Qiuyan Liao et al. "Effectiveness and Parental Acceptability of Social Networking Interventions for Promoting Seasonal Influenza Vaccination Among Young Children: Randomized Controlled Trial." In: *Journal of medical Internet research* 22.2 (Feb. 28, 2020), e16427. ISSN: 1438-8871 1439-4456. DOI: 10.2196/16427. pmid: 32130136.

[78] Tracy A. Lieu et al. "Effect of Electronic and Mail Outreach From Primary Care Physicians for COVID-19 Vaccination of Black and Latino Older Adults: A Randomized Clinical Trial." In: *JAMA network open* 5.6 (June 1, 2022), e2217004. ISSN: 2574-3805. DOI: 10.1001/jamanetworkopen.2022.17004. pmid: 35713906.

[79] Eleni Mantzari, Florian Vogt, and Theresa M. Marteau. "Financial Incentives for Increasing Uptake of HPV Vaccinations: A Randomized Controlled Trial." In: *Health psychology : official journal of the Division of Health Psychology, American Psychological Association* 34.2 (Feb. 2015), pp. 160–171. ISSN: 1930-7810 0278-6133. DOI: 10.1037/hea0000088. pmid: 25133822.

[80] Nell J. Marshall et al. "Influence of Digital Intervention Messaging on Influenza Vaccination Rates Among Adults With Cardiovascular Disease in the United States: Decentralized Randomized Controlled Trial." In: *Journal of medical Internet research* 24.10 (Oct. 7, 2022), e38710. ISSN: 1438-8871 1439-4456. DOI: 10.2196/38710. pmid: 36206046.

[81] Shivan J. Mehta et al. "Effect of Text Messaging and Behavioral Interventions on COVID-19 Vaccination Uptake: A Randomized Clinical Trial." In: *JAMA network open* 5.6 (June 1, 2022), e2216649. ISSN: 2574-3805. DOI: 10.1001/jamanetworkopen.2022.16649. pmid: 35696165.

[82] Zeleke Abebaw Mekonnen et al. "Effect of Mobile Phone Text Message Reminders on the Completion and Timely Receipt of Routine Childhood Vaccinations: Superiority Randomized Controlled Trial in Northwest Ethiopia." In: *JMIR mHealth and uHealth* 9.6 (June 15, 2021), e27603. ISSN: 2291-5222. DOI: 10.2196/27603. pmid: 34128813.

[83] Katherine L. Milkman et al. "A 680,000-Person Megastudy of Nudges to Encourage Vaccination in Pharmacies". In: *Proceedings of the National Academy of Sciences* 119.6 (Feb. 8, 2022), e2115126119. ISSN: 0027-8424, 1091-6490. DOI: 10.1073/pnas.2115126119. URL: https://pnas.org/doi/full/10.1073/pnas.2115126119 (visited on 01/16/2023).

[84] Katherine L. Milkman et al. "A Citywide Experiment Testing the Impact of Geographically Targeted, High-Pay-off Vaccine Lotteries". In: *Nature Human Behaviour* 6.11 (Sept. 1, 2022), pp. 1515–1524. ISSN: 2397-3374. DOI: 10.1038/s41562-022-01437-0. URL: https://www.nature.com/articles/s41562-022-01437-0 (visited on 01/16/2023).

[85] Katherine L. Milkman et al. "A Megastudy of Text-Based Nudges Encouraging Patients to Get Vaccinated at an Upcoming Doctor's Appointment". In: *Proceedings of the National Academy of Sciences* 118.20 (May 18, 2021), e2101165118. ISSN: 0027-8424, 1091-6490. DOI: 10.1073/pnas.2101165118. URL: https://pnas.org/doi/full/10.1073/pnas.2101165118 (visited on 01/16/2023).

[86] Katherine L. Milkman et al. "Using Implementation Intentions Prompts to Enhance Influenza Vaccination Rates". In: *Proceedings of the National Academy of Sciences* 108.26 (June 28, 2011), pp. 10415–10420. ISSN: 0027-8424, 1091-6490. DOI: 10.1073/pnas.1103170108. URL: https://pnas.org/doi/full/10.1073/pnas.1103170108 (visited on 01/16/2023).

[87] Deborah S. Minor et al. "Improving Influenza Vaccination Rates by Targeting Individuals Not Seeking Early Seasonal Vaccination." In: *The American journal of medicine* 123.11 (Nov. 2010), pp. 1031–1035. ISSN: 1555-7162 0002-9343. DOI: 10.1016/j.amjmed.2010.06.017. pmid: 20843496.

[88] Michelle H. Moniz et al. “Improving Influenza Vaccination Rates in Pregnancy through Text Messaging: A Randomized Controlled Trial.” In: *Obstetrics and gynecology* 121.4 (Apr. 2013), pp. 734–740. ISSN: 1873-233X 0029-7844. DOI: 10.1097/AOG.0b013e31828642b1. pmid: 23635672.

[89] Ruchit Nagar et al. “A Cluster Randomized Trial to Determine the Effectiveness of a Novel, Digital Pendant and Voice Reminder Platform on Increasing Infant Immunization Adherence in Rural Udaipur, India.” In: *Vaccine* 36.44 (Oct. 22, 2018), pp. 6567–6577. ISSN: 1873-2518 0264-410X. DOI: 10.1016/j.vaccine.2017.11.023. pmid: 29162321.

[90] National Council of Applied Economic Research et al. *Impacts of the Stimulate, Appreciate, Learn and Transfer Community Engagement Approach to Increase Immunization Coverage in Assam, India.* International Initiative for Impact Evaluation (3ie), Dec. 2020. DOI: 10.23846/TW10IE130. URL: https://www.3ieimpact.org/evidence-hub/publications/impact-evaluations/impacts-stimulate-appreciate-learn-and-transfer (visited on 01/19/2023).

[91] Eileen K. Nehme et al. “Promoting Influenza Vaccination Among an ACA Health Plan Subscriber Population: A Randomized Trial.” In: *American journal of health promotion : AJHP* 33.6 (July 2019), pp. 916–920. ISSN: 2168-6602 0890-1171. DOI: 10.1177/0890117118823157. pmid: 30630342.

[92] Mary Patricia Nowalk et al. “Improving Influenza Vaccination Rates in the Workplace: A Randomized Trial.” In: *American journal of preventive medicine* 38.3 (Mar. 2010), pp. 237–246. ISSN: 1873-2607 0749-3797. DOI: 10.1016/j.amepre.2009.11.011. pmid: 20036102.

[93] Mary Patricia Nowalk et al. “Increasing Childhood Influenza Vaccination: A Cluster Randomized Trial.” In: *American journal of preventive medicine* 47.4 (Oct. 2014), pp. 435–443. ISSN: 1873-2607 0749-3797. DOI: 10.1016/j.amepre.2014.07.003. pmid: 25113138.

[94] Kerry-Ann F. O’Grady et al. “SMS Reminders to Improve the Uptake and Timeliness of the Primary Immunisation Series in Infants: A Multi-Centre Randomised Controlled Trial.” In: *Communicable diseases intelligence (2018)* 46 (May 19, 2022). ISSN: 2209-6051. DOI: 10.33321/cdi.2022.46.15. pmid: 35591748.

[95] Sean T. O’Leary et al. “Effectiveness and Cost of Bidirectional Text Messaging for Adolescent Vaccines and Well Care”. In: *Pediatrics* 136.5 (Nov. 1, 2015), e1220–e1227. ISSN: 0031-4005, 1098-4275. DOI: 10.1542/peds.2015-1089. URL: https://publications.aap.org/pediatrics/article/136/5/e1220/33786/Effectiveness-and-Cost-of-Bidirectional-Text (visited on 01/16/2023).

[96] Sean T. O'Leary et al. "Effectiveness of a Multimodal Intervention to Increase Vaccination in Obstetrics/Gynecology Settings." In: *Vaccine* 37.26 (June 6, 2019), pp. 3409–3418. ISSN: 1873-2518 0264-410X. DOI: 10.1016/j.vaccine.2019.05.034. pmid: 31103367.

[97] Sean T. O'Leary et al. "Efficacy of a Web-Based Intervention to Increase Uptake of Maternal Vaccines: An RCT." In: *American journal of preventive medicine* 57.4 (Oct. 2019), e125–e133. ISSN: 1873-2607 0749-3797. DOI: 10.1016/j.amepre.2019.05.018. pmid: 31471001.

[98] Aatekah Owais et al. "Does Improving Maternal Knowledge of Vaccines Impact Infant Immunization Rates? A Community-Based Randomized-Controlled Trial in Karachi, Pakistan." In: *BMC public health* 11 (Apr. 17, 2011), p. 239. ISSN: 1471-2458. DOI: 10.1186/1471-2458-11-239. pmid: 21496343.

[99] Angela Oyo-Ita et al. "Effects of Engaging Communities in Decision-Making and Action through Traditional and Religious Leaders on Vaccination Coverage in Cross River State, Nigeria: A Cluster-Randomised Control Trial." In: *PloS one* 16.4 (2021), e0248236. ISSN: 1932-6203. DOI: 10.1371/journal.pone.0248236. pmid: 33861742.

[100] Priyanka Pandey et al. "Informing Resource-Poor Populations and the Delivery of Entitled Health and Social Services in Rural India: A Cluster Randomized Controlled Trial." In: *JAMA* 298.16 (Oct. 24, 2007), pp. 1867–1875. ISSN: 1538-3598 0098-7484. DOI: 10.1001/jama.298.16.1867. pmid: 17954538.

[101] Ashlesha Patel et al. "Staying on Track: A Cluster Randomized Controlled Trial of Automated Reminders Aimed at Increasing Human Papillomavirus Vaccine Completion." In: *Vaccine* 32.21 (May 1, 2014), pp. 2428–2433. ISSN: 1873-2518 0264-410X. DOI: 10.1016/j.vaccine.2014.02.095. pmid: 24631099.

[102] Mitesh S. Patel et al. "Effect of Text Message Reminders and Vaccine Reservations on Adherence to a Health System COVID-19 Vaccination Policy: A Randomized Clinical Trial." In: *JAMA network open* 5.7 (July 1, 2022), e2222116. ISSN: 2574-3805. DOI: 10.1001/jamanetworkopen.2022.22116. pmid: 35857327.

[103] Timothy Powell-Jackson et al. "Effect and Cost-Effectiveness of Educating Mothers about Childhood DPT Vaccination on Immunisation Uptake, Knowledge, and Perceptions in Uttar Pradesh, India: A Randomised Controlled Trial." In: *PLoS medicine* 15.3 (Mar. 2018), e1002519. ISSN: 1549-1676 1549-1277. DOI: 10.1371/journal.pmed.1002519. pmid: 29509769.

[104] Wei Qin et al. "Upgrading the School Entry Vaccination Record Check Strategy to Improve Varicella Vaccination Coverage: Results from a Quasi-Experiment Study." In: *Human vaccines & immunotherapeutics* 17.9 (Sept. 2, 2021), pp. 3137–3144. ISSN: 2164-554X 2164-5515. DOI: 10.1080/21645515.2021.1904759. pmid: 34019470.

[105] Nathaniel Rabb et al. "Evidence from a Statewide Vaccination RCT Shows the Limits of Nudges". In: *Nature* 604.7904 (Apr. 7, 2022), E1–E7. ISSN: 0028-0836, 1476-4687. DOI: 10.1038/s41586-022-04526-2. URL: https://www.nature.com/articles/s41586-022-04526-2 (visited on 01/16/2023).

[106] Annette K. Regan et al. "Randomized Controlled Trial of Text Message Reminders for Increasing Influenza Vaccination." In: *Annals of family medicine* 15.6 (Nov. 2017), pp. 507–514. ISSN: 1544-1717 1544-1709. DOI: 10.1370/afm.2120. pmid: 29133488.

[107] Bernardino Roca et al. "Impact of Education Program on Influenza Vaccination Rates in Spain." In: *The American journal of managed care* 18.12 (Dec. 1, 2012), e446–452. ISSN: 1936-2692 1088-0224. pmid: 23286674.

[108] D. Santa Maria et al. "Effects of a Randomized Controlled Trial of a Brief, Student-Nurse Led, Parent-Based Sexual Health Intervention on Parental Protective Factors and HPV Vaccination Uptake." In: *BMC public health* 21.1 (Mar. 24, 2021), p. 585. ISSN: 1471-2458. DOI: 10.1186/s12889-021-10534-0. pmid: 33761920.

[109] Ryoko Sato and Yoshito Takasaki. "Backfire Effect of Salient Information on Vaccine Take-up Experimental Evidence from Scared-Straight Intervention in Rural Northern Nigeria." In: *Human vaccines & immunotherapeutics* 17.6 (June 3, 2021), pp. 1703–1713. ISSN: 2164-554X 2164-5515. DOI: 10.1080/21645515.2020.1836917. pmid: 33325768.

[110] Lauri Sääksvuori et al. "Information Nudges for Influenza Vaccination: Evidence from a Large-Scale Cluster-Randomized Controlled Trial in Finland." In: *PLoS medicine* 19.2 (Feb. 2022), e1003919. ISSN: 1549-1676 1549-1277. DOI: 10.1371/journal.pmed.1003919. pmid: 35139082.

[111] Paramita Sengupta et al. "Evaluation of a Community-Based Intervention to Improve Routine Childhood Vaccination Uptake among Migrants in Urban Slums of Ludhiana, India." In: *Journal of public health (Oxford, England)* 39.4 (Dec. 1, 2017), pp. 805–812. ISSN: 1741-3850 1741-3842. DOI: 10.1093/pubmed/fdw131. pmid: 27915261.

[112] Mingyu Si et al. "An Internet-Based Education Program for Human Papillomavirus Vaccination Among Female College Students in Mainland China: Application of the Information-Motivation-Behavioral Skills Model in a Cluster Randomized Trial." In: *Journal of medical Internet research* 24.9 (Sept. 30, 2022), e37848. ISSN: 1438-8871 1439-4456. DOI: 10.2196/37848. pmid: 36178723.

[113] Danya Arif Siddiqi et al. "Effect of Vaccine Reminder and Tracker Bracelets on Routine Childhood Immunization Coverage and Timeliness in Urban Pakistan (2017-18): A Randomized Controlled Trial." In: *BMC public health* 20.1 (July 11, 2020), p. 1086. ISSN: 1471-2458. DOI: 10.1186/s12889-020-09088-4. pmid: 32652969.

[114] A. Niroshan Siriwardena et al. "Cluster Randomised Controlled Trial of an Educational Outreach Visit to Improve Influenza and Pneumococcal Immunisation Rates in Primary Care." In: *The British journal of general practice : the journal of the Royal College of General Practitioners* 52.482 (Sept. 2002), pp. 735–740. ISSN: 0960-1643. pmid: 12236277.

[115] S. R. Skinner et al. "Randomised Controlled Trial of an Educational Strategy to Increase School-Based Adolescent Hepatitis B Vaccination." In: *Australian and New Zealand journal of public health* 24.3 (June 2000), pp. 298–304. ISSN: 1326-0200. DOI: 10.1111/j.1467-842x.2000.tb01572.x. pmid: 10937408.

[116] Melissa S. Stockwell et al. "Text Message Reminders for Second Dose of Influenza Vaccine: A Randomized Controlled Trial." In: *Pediatrics* 135.1 (Jan. 2015), e83–91. ISSN: 1098-4275 0031-4005. DOI: 10.1542/peds.2014-2475. pmid: 25548329.

[117] Samuel Stolpe and Niteesh K. Choudhry. "Effect of Automated Immunization Registry-Based Telephonic Interventions on Adult Vaccination Rates in Community Pharmacies: A Randomized Controlled Trial." In: *Journal of managed care & specialty pharmacy* 25.9 (Sept. 2019), pp. 989–994. ISSN: 2376-1032. DOI: 10.18553/jmcp.2019.25.9.989. pmid: 31456496.

[118] Christina A. Suh et al. "Effectiveness and Net Cost of Reminder/Recall for Adolescent Immunizations." In: *Pediatrics* 129.6 (June 2012), e1437–1445. ISSN: 1098-4275 0031-4005. DOI: 10.1542/peds.2011-1714. pmid: 22566415.

[119] Peter Szilagyi et al. "Effect of State Immunization Information System Centralized Reminder and Recall on HPV Vaccination Rates." In: *Pediatrics* 145.5 (May 2020). ISSN: 1098-4275 0031-4005. DOI: 10.1542/peds.2019-2689. pmid: 32253263.

[120] Peter G. Szilagyi et al. "A Randomized Trial of the Effect of Centralized Reminder/Recall on Immunizations and Preventive Care Visits for Adolescents." In: *Academic pediatrics* 13.3 (2013 May-Jun), pp. 204–213. ISSN: 1876-2867 1876-2859. DOI: 10.1016/j.acap.2013.01.002. pmid: 23510607.

[121] Peter G. Szilagyi et al. "Effect of Training Pediatric Clinicians in Human Papillomavirus Communication Strategies on Human Papillomavirus Vaccination Rates: A Cluster Randomized Clinical Trial." In: *JAMA pediatrics* 175.9 (Sept. 1, 2021), pp. 901–910. ISSN: 2168-6211 2168-6203. DOI: 10.1001/jamapediatrics.2021.0766. pmid: 34028494.

[122] Peter G. Szilagyi et al. "Effectiveness of a Citywide Patient Immunization Navigator Program on Improving Adolescent Immunizations and Preventive Care Visit Rates." In: *Archives of pediatrics & adolescent medicine* 165.6 (June 2011), pp. 547–553. ISSN: 1538-3628 1072-4710. DOI: 10.1001/archpediatrics.2011.73. pmid: 21646588.

[123] Katya Tentori et al. "Nudging COVID-19 Vaccine Uptake by Changing the Default: A Randomized Controlled Trial." In: *Medical decision making : an international journal of the Society for Medical Decision Making* 42.6 (Aug. 2022), pp. 837–841. ISSN: 1552-681X 0272-989X. DOI: 10.1177/0272989X221101536. pmid: 35658775.

[124] Jasmin A. Tiro et al. "Promoting HPV Vaccination in Safety-Net Clinics: A Randomized Trial." In: *Pediatrics* 136.5 (Nov. 2015), pp. 850–859. ISSN: 1098-4275 0031-4005. DOI: 10.1542/peds.2015-1563. pmid: 26482674.

[125] Libby Topp et al. "A Randomised Controlled Trial of Financial Incentives to Increase Hepatitis B Vaccination Completion among People Who Inject Drugs in Australia." In: *Preventive medicine* 57.4 (Oct. 2013), pp. 297–303. ISSN: 1096-0260 0091-7435. DOI: 10.1016/j.ypmed.2013.04.013. pmid: 23639625.

[126] Fraser Tull et al. "Short Message Service Reminders to Parents for Increasing Adolescent Human Papillomavirus Vaccination Rates in a Secondary School Vaccine Program: A Randomized Control Trial." In: *The Journal of adolescent health : official publication of the Society for Adolescent Medicine* 65.1 (July 2019), pp. 116–123. ISSN: 1879-1972 1054-139X. DOI: 10.1016/j.jadohealth.2018.12.026. pmid: 30879881.

[127] Md. Jasim Uddin et al. "Use of Mobile Phones for Improving Vaccination Coverage among Children Living in Rural Hard-to-Reach Areas and Urban Streets of Bangladesh". In: *Vaccine* 34.2 (Jan. 2016), pp. 276–283. ISSN: 0264410X. DOI: 10.1016/j.vaccine.2015.11.024. URL: https://linkinghub.elsevier.com/retrieve/pii/S0264410X15016667 (visited on 01/16/2023).

[128] Natasha L. Underwood et al. "Evaluation of Educational Interventions to Enhance Adolescent Specific Vaccination Coverage." In: *The Journal of school health* 89.8 (Aug. 2019), pp. 603–611. ISSN: 1746-1561 0022-4391. DOI: 10.1111/josh.12786. pmid: 31161606.

[129] Takashi Usami et al. "Impact of Community Pharmacists Advocating Immunization on Influenza Vaccination Rates among the Elderly." In: *Yakugaku zasshi : Journal of the Pharmaceutical Society of Japan* 129.9 (Sept. 2009), pp. 1063–1068. ISSN: 0031-6903. DOI: 10.1248/yakushi.129.1063. pmid: 19721382.

[130] Hussain R. Usman et al. "Randomized Controlled Trial to Improve Childhood Immunization Adherence in Rural Pakistan: Redesigned Immunization Card and Maternal Education." In: *Tropical medicine & international health : TM & IH* 16.3 (Mar. 2011), pp. 334–342. ISSN: 1365-3156 1360-2276. DOI: 10.1111/j.1365-3156.2010.02698.x. pmid: 21159080.

[131] P. M. Vivier et al. "The Impact of Outreach Efforts in Reaching Underimmunized Children in a Medicaid Managed Care Practice." In: *Archives of pediatrics & adolescent medicine* 154.12 (Dec. 2000), pp. 1243–1247. ISSN: 1072-4710. DOI: 10.1001/archpedi.154.12.1243. pmid: 11115310.

[132] Nicole M. Wagner et al. "Addressing Logistical Barriers to Childhood Vaccination Using an Automated Reminder System and Online Resource Intervention: A Randomized Controlled Trial." In: *Vaccine* 39.29 (June 29, 2021), pp. 3983–3990. ISSN: 1873-2518 0264-410X. DOI: 10.1016/j.vaccine.2021.05.053. pmid: 34059372.

[133] Tim Weaver et al. "Use of Contingency Management Incentives to Improve Completion of Hepatitis B Vaccination in People Undergoing Treatment for Heroin Dependence: A Cluster Randomised Trial." In: *Lancet (London, England)* 384.9938 (July 12, 2014), pp. 153–163. ISSN: 1474-547X 0140-6736. DOI: 10.1016/S0140-6736(14)60196-3. pmid: 24725468.

[134] Jennifer A. Whitaker et al. "Immunization Education for Internal Medicine Residents: A Cluster-Randomized Controlled Trial." In: *Vaccine* 36.14 (Mar. 27, 2018), pp. 1823–1829. ISSN: 1873-2518 0264-410X. DOI: 10.1016/j.vaccine.2018.02.082. pmid: 29496350.

[135] Jessica G. Wijesundara et al. "Electronic Health Record Portal Messages and Interactive Voice Response Calls to Improve Rates of Early Season Influenza Vaccination: Randomized Controlled Trial." In: *Journal of medical Internet research* 22.9 (Sept. 25, 2020), e16373. ISSN: 1438-8871 1439-4456. DOI: 10.2196/16373. pmid: 32975529.

[136] S. Elizabeth Williams, Laura E. Adams, and Evan C. Sommer. "Improving Vaccination for Young Children (IVY): A Stepped-Wedge Cluster Randomized Trial." In: *Academic pediatrics* 21.7 (2021 Sep-Oct), pp. 1151–1160. ISSN: 1876-2867 1876-2859. DOI: 10.1016/j.acap.2021.06.001. pmid: 34118498.

[137] Carla A. Winston, Adrienne D. Mims, and Kecia A. Leatherwood. "Increasing Pneumococcal Vaccination in Managed Care through Telephone Outreach". In: *The American Journal of Managed Care* 13.10 (Oct. 2007), pp. 581–588. ISSN: 1936-2692. pmid: 17927463.

[138] Valerie Wing Yu Wong et al. "Brief Education to Promote Maternal Influenza Vaccine Uptake: A Randomized Controlled Trial." In: *Vaccine* 34.44 (Oct. 17, 2016), pp. 5243–5250. ISSN: 1873-2518 0264-410X. DOI: 10.1016/j.vaccine.2016.09.019. pmid: 27667330.

[139] Karene Hoi Ting Yeung et al. "Increasing Influenza Vaccine Uptake in Children: A Randomised Controlled Trial." In: *Vaccine* 36.37 (Sept. 5, 2018), pp. 5524–5535. ISSN: 1873-2518 0264-410X. DOI: 10.1016/j.vaccine.2018.07.066. pmid: 30078745.

[140] David Yokum et al. "Letters Designed with Behavioural Science Increase Influenza Vaccination in Medicare Beneficiaries". In: *Nature Human Behaviour* 2.10 (Oct. 1, 2018), pp. 743–749. ISSN: 2397-3374. DOI: 10.1038/s41562-018-0432-2. URL: https://www.nature.com/articles/s41562-018-0432-2 (visited on 01/16/2023).

[141] Byung-Kwang Yoo et al. "Cost Effectiveness Analysis of Year 2 of an Elementary School-Located Influenza Vaccination Program–Results from a Randomized Controlled Trial". In: *BMC Health Services Research* 15.1 (June 2015), p. 511. ISSN: 1472-6963. DOI: 10.1186/s12913-015-1169-5. URL: http://bmchealthservres.biomedcentral.com/articles/10.1186/s12913-015-1169-5 (visited on 01/16/2023).

[142] Mark H. Yudin et al. "Text Messages for Influenza Vaccination among Pregnant Women: A Randomized Controlled Trial." In: *Vaccine* 35.5 (Feb. 1, 2017), pp. 842–848. ISSN: 1873-2518 0264-410X. DOI: 10.1016/j.vaccine.2016.12.002. pmid: 28062124.

[143] Mu Yue et al. "Optimal Design of Population-Level Financial Incentives of Influenza Vaccination for the Elderly." In: *Value in health : the journal of the International Society for Pharmacoeconomics and Outcomes Research* 23.2 (Feb. 2020), pp. 200–208. ISSN: 1524-4733 1098-3015. DOI: 10.1016/j.jval.2019.08.006. pmid: 32113625.

[144] Richard K. Zimmerman et al. "Improving Adolescent HPV Vaccination in a Randomized Controlled Cluster Trial Using the 4 Pillars™ Practice Transformation Program." In: *Vaccine* 35.1 (Jan. 3, 2017), pp. 109–117. ISSN: 1873-2518 0264-410X. DOI: 10.1016/j.vaccine.2016.11.018. pmid: 27876200.
