# Supplementary material for: Last-mile delivery increases vaccine uptake in Sierra Leone
Source: Nature. 2024 Mar 13;627(8004):612–9. doi: 10.1038/s41586-024-07158-w (PMC10954551; doi:10.1038/s41586-024-07158-w)
Supplement: Supplementary file 2 — Reporting Summary [file 41586_2024_7158_MOESM2_ESM.pdf]

nature portfolio

Corresponding author(s): A. M. Mobarak

Last updated by author(s): Nov 7, 2023

Reporting Summary

Nature Portfolio wishes to improve the reproducibility of the work that we publish. This form provides structure for consistency and transparency in reporting. For further information on Nature Portfolio policies, see our Editorial Policies and the Editorial Policy Checklist.

Statistics

For all statistical analyses, confirm that the following items are present in the figure legend, table legend, main text, or Methods section.

n/a	Confirmed	
☐	☒	The exact sample size (n) for each experimental group/condition, given as a discrete number and unit of measurement
☐	☒	A statement on whether measurements were taken from distinct samples or whether the same sample was measured repeatedly
☐	☒	The statistical test(s) used AND whether they are one- or two-sided<br>*Only common tests should be described solely by name; describe more complex techniques in the Methods section.*
☐	☒	A description of all covariates tested
☐	☒	A description of any assumptions or corrections, such as tests of normality and adjustment for multiple comparisons
☐	☒	A full description of the statistical parameters including central tendency (e.g. means) or other basic estimates (e.g. regression coefficient) AND variation (e.g. standard deviation) or associated estimates of uncertainty (e.g. confidence intervals)
☐	☒	For null hypothesis testing, the test statistic (e.g. F, t, r) with confidence intervals, effect sizes, degrees of freedom and P value noted<br>*Give P values as exact values whenever suitable.*
☒	☐	For Bayesian analysis, information on the choice of priors and Markov chain Monte Carlo settings
☒	☐	For hierarchical and complex designs, identification of the appropriate level for tests and full reporting of outcomes
☐	☒	Estimates of effect sizes (e.g. Cohen's d, Pearson's r), indicating how they were calculated

Our web collection on statistics for biologists contains articles on many of the points above.

Software and code

Policy information about availability of computer code

Data collection	For primary data collection we used ODK survey collection software, version 2.81
Data analysis	For our data analysis we STATA SE 17

For manuscripts utilizing custom algorithms or software that are central to the research but not yet described in published literature, software must be made available to editors and reviewers. We strongly encourage code deposition in a community repository (e.g. GitHub). See the Nature Portfolio guidelines for submitting code & software for further information.

Data

Policy information about availability of data

All manuscripts must include a data availability statement. This statement should provide the following information, where applicable:

- Accession codes, unique identifiers, or web links for publicly available datasets
- A description of any restrictions on data availability
- For clinical datasets or third party data, please ensure that the statement adheres to our policy

Primary survey data was collected for the research and can be made available to reviewers.
All survey will be de-identified and deposited in Harvard Dataverse upon publication.

Research involving human participants, their data, or biological material

Policy information about studies with human participants or human data. See also policy information about sex, gender (identity/presentation), and sexual orientation and race, ethnicity and racism.

Reporting on sex and gender	We include subgroup analysis by gender (as self reported by respondents) in Figure 5 and Extended Data Table 8
Reporting on race, ethnicity, or other socially relevant groupings	We also provide subgroup analysis by education (any education, or none), age group (18-25, 25-54, and older) and land ownership (whether the household owns any land), food security (whether households reduced portions of food) in in Figure 5 and Extended Data Table 8
Population characteristics	The population of these villages was on average 22.3 years old, 26.5% of households were female-headed, 64.7% of people lived in a household of 6 or fewer people. Just 20.2% lived in a household where the household head had any form of formal schooling, and about 86.2% lived in a household where the head was primarily engaged in farming.
Recruitment	Participant inclusion criteria for the intervention: All residents of selected rural communities eligible for the COVID-19 vaccine in 150 communities selected from seven rural districts in Sierra Leone (Koinadugu, Falaba, Karene, Kambia, Tonkolili, Bombali and Port Loko). For recruitment into the survey, see Sampling Strategy below.
Ethics oversight	We received Institutional Review Board (IRB) approval from the Sierra Leone Ethics and Scientific Review Committee (SLERC 20220210), Yale University (2000031541) and Wageningen University (WUR 20220222). The research protocol was pre-registered at ISRCTN (study ISRCTN 17878735, see https://doi.org/10.1186/ISRCTN17878735). All study participants completed informed consent.

Note that full information on the approval of the study protocol must also be provided in the manuscript.

Field-specific reporting

Please select the one below that is the best fit for your research. If you are not sure, read the appropriate sections before making your selection.

☐ Life sciences ☒ Behavioural & social sciences ☐ Ecological, evolutionary & environmental sciences

For a reference copy of the document with all sections, see nature.com/documents/nr-reporting-summary-flat.pdf

Behavioural & social sciences study design

All studies must disclose on these points even when the disclosure is negative.

Study description	We conduct a randomized control trial where we study the impact of an intervention increasing access to vaccines (specifically COVID-19 vaccines) and two different mobalisation strategies on vaccine uptake in rural communities in Sierra Leone, a lower income country characterized by a poor health infrastructure. We find that our intervention increased vaccine uptake by over 25 pp and can be implemented quickly and cost effectively. These features make our intervention particularly suited for situations where a quick vaccination roll out is needed in places that are severely resource constrained.
Research sample	The objective of the study was to learn about last mile challenges in vaccine uptake in Low Income Countries. The sample consists of 150 rural and remote villages in 7 districts (Koinadugu, Falaba, Karene, Kambia, Tonkolili, Bombali, Port Loko) in Sierra Leone. Villages lie outside the regular catchment areas of health clinics, where access to vaccines are is low, -- ie “hard cases”. The majority of sub-Saharan Africans reside in rural areas, and face similar challenges as our study sample.
Sampling strategy	We used a power calculation to determine the number of villages needed for a range of minimum detectable effect sizes assuming a 5% significance level with 80% power. We expected decisions to take a vaccine should be highly correlated within a village, so we assumed an ICC of 0.15. In the 2015 census data, the mean population is 2480 people per village. Assuming that 50% of them were eligible to take the vaccine, we assumed an effective village size of 1200 people per village. Given that vaccine take up in Sierra Leone is low, we assumed a baseline vaccination rate of 2.5%. Based on the treatment effects reported in the literature for similar studies and the large heterogeneity in treatment effects reported, we adopt a conservative approach and set our expected MDE at 0.05. We oversampled slightly and the final design included 150 communities across the three treatment arms, in 1:1:1 ratio.<br><br>Village level sample: we used the 2015 Sierra Leone Census and selected 7 largely rural districts (Koinadugu, Falaba, Karene, Kambia, Tonkolili, Bombali, Port Loko) with 8,784 communities in 54 Chiefdoms. We then restricted the sample to communities with no health clinic within five miles of the community center, resulting in 1,849 communities. We further excluded very small communities (below 19 structures) and for which latitude and longitude was missing. The final sampling frame of 420 communities were located in 49 Chiefdoms and 7 Districts. Within each District, we then matched communities on the following strata: i. the share of the population that was immunized, ii. the age of the population, iii. literacy levels, and the iv. distance from the closest clinic. This allowed us to identify communities that had the most similar characteristics within a district and used this to assign the most similar communities to one of the treatment group and establish comparable "triplets". This resulted in 106 triplets in total. We then randomly selected 50 triplets (and 150 villages) using district as a blocking variable. The final list included: 9 triplets each for Koinadugu and Falaba District, 8 triplets for Karene District and 6 triplets each for Port Loko, Tonkolili, Kambia and Bombali District. Within each village we used several instruments collect data:<br>• Census with all household heads, going door to door, to help make a roster of those living under the same roof and eating from the

	same pot.<br>• Baseline household survey: n all communities, we randomly selected a sample of 30 households per village from the census/listing exercise<br>• Exit survey: of those that took a vaccine taken at each mobile vaccination site<br>• Endline household survey: with the same households that we interviewed at baseline
Data collection	Data was collected using a standardized questionnaire on tablets using SurveyCTO software. Questionnaires were conducted by local research assistants hired by the research team, using electronic tablets to record survey responses. Besides these research assistants, no one from the research team or from the implementing partner was present during the questionnaires. The research assistants were trained to conduct the questionnaire but were blinded to the research questions of this study. Other aspects of training including following important ethical guidelines, maintaining neutrality, and ensuring the respondents' comfort during the process. High frequency checks were implemented throughout the data collection process. The research assistants' work was routinely audited to ensure consistent adherence to the standardized questionnaire and data collection protocols. Besides quality checks, survey data was stored in a password-protected server, and was only accessible to the authors of this submission.
Timing	Our survey data were collected, and the accompanying interventions undertaken, during March and April 2022.
Data exclusions	No data were excluded from analysis.
Non-participation	All 150 village remained in the study throughout. At the respondent level: for the baseline 4 respondents, endline 0 respondents and for the exit survey 6 respondents declined participation. No reasons were for non consent were provided.
Randomization	We used blocked randomisation: within each block of three villages we assigned villages to either a control group, "door to door" treatment, or "small group" treatment arm. in a 1:1:1 ratio. This resulted in 50 villages assigned to control, 50 to "door to door", and 50 to "small group". Within the "door to door" villages, we randomly assigned randomly selected 20 residential structures from the community census list to receive a visit from the social mobilisation team.

Reporting for specific materials, systems and methods

We require information from authors about some types of materials, experimental systems and methods used in many studies. Here, indicate whether each material, system or method listed is relevant to your study. If you are not sure if a list item applies to your research, read the appropriate section before selecting a response.

Materials & experimental systems

n/a	Involved in the study	
☒	☐	Antibodies
☒	☐	Eukaryotic cell lines
☒	☐	Palaeontology and archaeology
☒	☐	Animals and other organisms
☒	☐	Clinical data
☒	☐	Dual use research of concern
☒	☐	Plants

Methods

n/a	Involved in the study	
☒	☐	ChIP-seq
☒	☐	Flow cytometry
☒	☐	MRI-based neuroimaging
